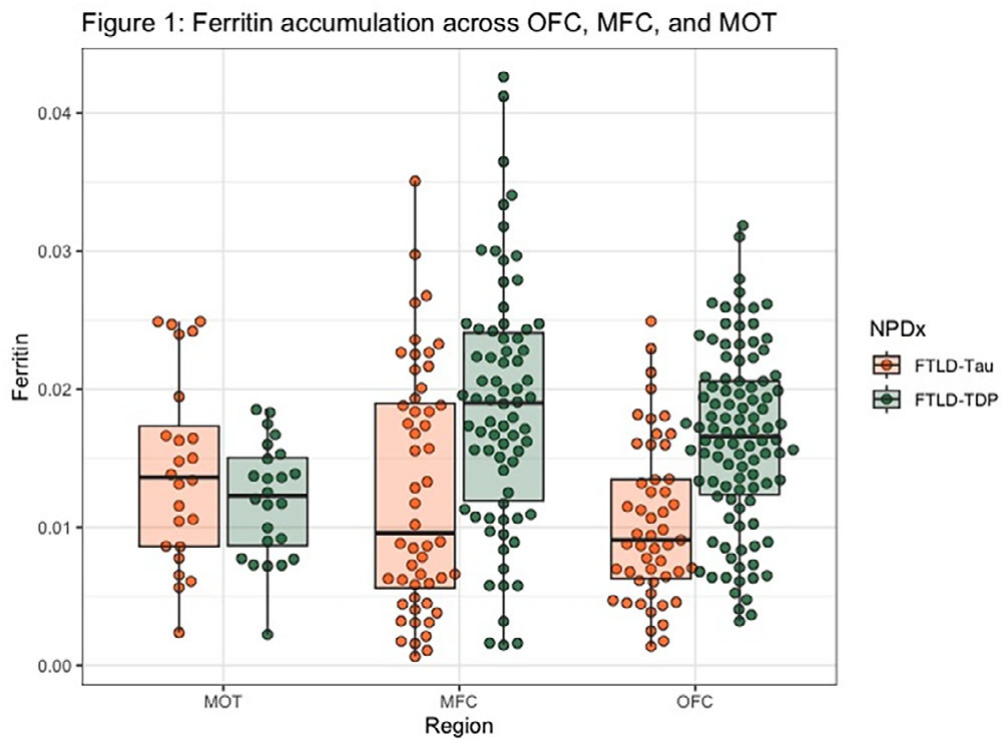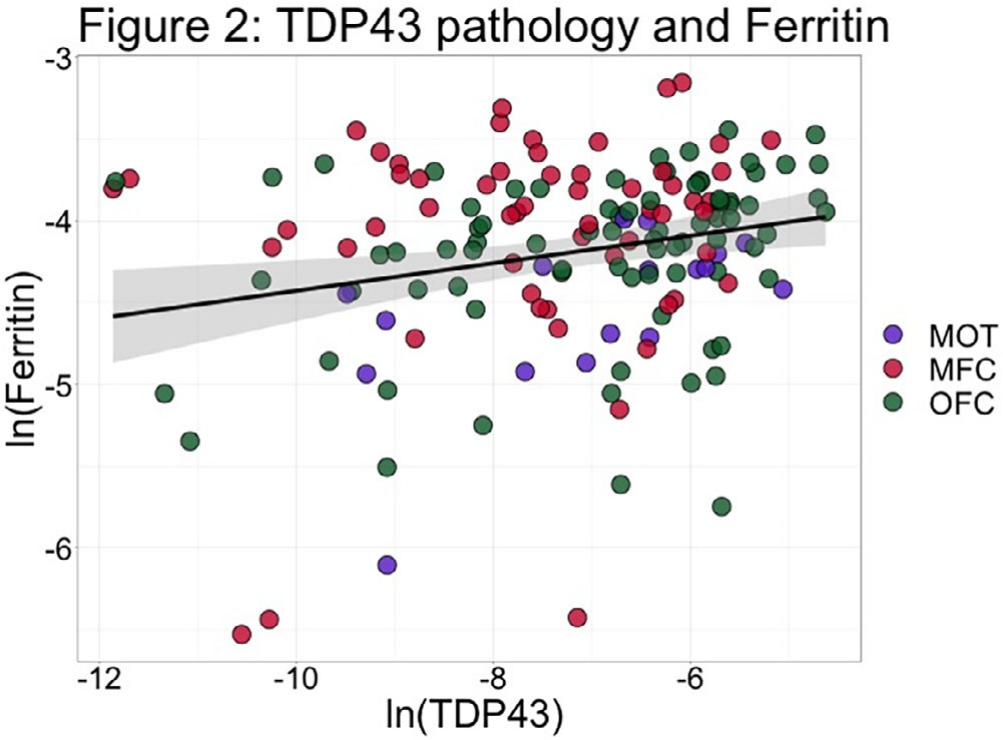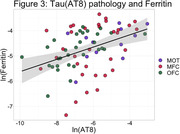# Patterns of iron‐rich gliosis in FTLD‐TDP versus FTLD‐Tau

**DOI:** 10.1002/alz.092610

**Published:** 2025-01-03

**Authors:** Sheina Emrani, Sanaz Arezoumandan, Katheryn A Q Cousins, Noah Capp, Eddie B Lee, David A Wolk, Paul A. Yushkevich, Corey T McMillan, M. Dylan Tisdall, David J Irwin

**Affiliations:** ^1^ Department of Neurology, Perelman School of Medicine, University of Pennsylvania, Philadelphia, PA USA; ^2^ Department of Neurology, University of Pennsylvania, Philadelphia, PA USA; ^3^ Department of Pathology & Laboratory Medicine, University of Pennsylvania, Philadelphia, PA USA; ^4^ Center for Neurodegenerative Disease Research, University of Pennsylvania, Philadelphia, PA USA; ^5^ Department of Pathology and Laboratory Medicine, Alzheimer’s Disease Center, Perelman School of Medicine, University of Pennsylvania, Philadelphia, PA USA; ^6^ University of Pennsylvania, Philadelphia, PA USA

## Abstract

**Background:**

We recently found region‐specific patterns of iron‐rich gliosis in frontotemporal lobar degeneration (FTLD) groups with tau (FTLD‐Tau; PSP) and TDP‐43 (FTLD‐TDP) pathology using iron‐sensitive MRI of whole‐hemispheres. These patterns largely corresponded to regions of early pathology reported in previous traditional histopathologic staging schemes of protein inclusions for FTLD‐Tau and FTLD‐TDP. Ferritin light chain (FLC) reactivity highlights activated glia but has not been studied extensively in FTLD. We hypothesize neuroinflammation marked by FLC‐reactive glia may relate to protein aggregation and contribute to partially distinct regional patterns of Tau vs TDP‐43 propagation in FTLD.

**Methods:**

We used digital histopathology to measure FLC % area occupied (%AO) in a pilot cohort of 48 FLTD‐Tau and 83 FTLD‐TDP patients in three frontal cortical regions implicated early in FTLD‐Tau (i.e., motor cortex [MOT]) and FTLD‐TDP (orbitofrontal cortex [OFC]) staging and an intermediate region for both staging schemes (medial frontal cortex [MFC]). We used linear mixed effect (LME) models to test the effect of region*group on FLC %AO while adjusting for age, sex, disease duration, and hemisphere sampled; individual was included as random‐intercept. Similarly, within‐group LME models tested for the association of %AO of Tau/TDP inclusions on FLC %AO. Between‐ and within‐group t‐tests were used to further examine FLC %AO levels.

**Results:**

In the total cohort, we found lower FLC%AO in MFC (Beta = ‐.008, SE = .002, p = <.001) and OFC (Beta = ‐.008 SE = .002, p = <.001) than MOT of FTLD‐Tau compared to FTLD‐TDP (Figure 1). T‐tests showed OFC FLC%AO was higher in FTLD‐TDP than FTLD‐Tau (p = <.001; Figure 1) and MOT FLC%AO was higher than OFC FLC%AO in FTLD‐Tau (p = 0.01). Separate LME models for Tau and TDP %AO was positively associated with FLC%AO across regions (Tau; Beta = 0.1 SE = .06, p = .03; TDP; Beta = 0.5 SE = .2, p = .02).

**Conclusion:**

In our preliminary sampling, iron‐rich gliosis is associated with protein aggregation in partially‐dissociated regional patterns in FLTD‐Tau vs FTLD‐TDP, recapitulating previous histopathological staging schemes. Future work will more comprehensively model the association of iron‐rich gliosis and protein aggregation across the brain.